# Dry Powder and Budesonide Inhalation Suspension Deposition Rates in Asthmatic Airway-Obstruction Regions

**DOI:** 10.1155/2019/3921426

**Published:** 2019-11-18

**Authors:** Norihide Murayama, Kei Asai, Kikuno Murayama, Satoru Doi, Makoto Kameda

**Affiliations:** ^1^Department of Pediatrics, Murayama Pediatrics, 3-2-33 Nagayoshi-Nagahara-Higashi Hirano-ku Osaka-shi, 547-0013 Osaka, Japan; ^2^Omron Healthcare Co. Ltd., 53 Kunotsubo, Terado-cho, Muko-shi, 617-0002 Kyoto, Japan; ^3^Department of Education, IBU Shitennnouji University, 3-2-1 Gakuenmae, Habikino, Osaka 583-8501, Japan; ^4^Department of Pediatrics, Osaka Habikino Medical Center, 3-7-1 Habikino Habikino-shi, Osaka, Japan

## Abstract

Steroid inhalation is the standard bronchial asthma therapy and it includes powdered metered doses, dry powder, and nebulizer suspension. However, particle sizes vary widely. The research goal was to demonstrate that different budesonide administration forms and devices have various deposition rates in the airway obstruction region. Here, we compared relative inhalation therapy efficacies and identified therapies that delivered the highest drug doses to the airway obstruction region. Weibel's anatomy data were used to identify the airway obstruction region in asthma. Based on European Standardization Committee data, we investigated the diameters of the drug particles being deposited there and evaluated the average particle size and distribution of the budesonide dosage forms and application devices. Drug dose depositions were measured by HPLC at each stage of a Cascade Impactor. Weibel's anatomy data indicated that the 1^st^–4^th^ bronchial generations comprised the airway obstruction region and corresponded to the tracheobronchial area. According to the European Standardization, particles 2–6 *µ*m in diameter were readily deposited there. The proportions of particles in this size range were 33.0%, 32.0%, 59.0%, and 78.0% for Turbuhaler, Symbicort, mesh-type NE-U22 suspension, and jet-type NE-C28 suspension, respectively. We localized the airway obstruction regions of bronchial asthma and identified the optimal inhalation therapy particle size. An electric nebulizer was more efficacious for budesonide administration than dry powder delivery. The NE-C28 treatment deposited 2.36x more budesonide in the airway obstruction region than dry powder delivery systems.

## 1. Introduction

Steroid inhalation therapy is the most popular treatment for both pediatric and adult bronchial asthma. Steroid inhalation is the most effective currently available therapy for bronchial asthma. It has other benefits such as inhibition of the development of serum-specific IgE [1] in childhood asthma. There are three main types of bronchial asthma inhalation methods: powdered metered dose inhalation (PMDI), dry powder inhalation (DPI), and electronic nebulizer suspensions (jet, ultrasonic, and mesh types). We propose that electronic nebulizer inhalation is the most effective of these three methods [2].

Bronchial asthma is obstructive and reacts to *β*2-stimulator disease without lung remodeling. To improve steroid inhalation efficacy, it is necessary to increase the amounts of steroids reaching the asthmatic obstruction regions. Certain factors such as steroid particle size (MMAD; mass median aerodynamic diameter), particle density, particle dosage form, particle surface tension, temperature, atmosphere, and airflow velocity affect steroid dose deposition. Among these factors, particle size is the most important because it significantly influences the deposition region.

Budesonide is marketed and supplied in two dosage forms. It is, therefore, possible to compare the two. The site and amount of inhaled antiasthma drug deposition are important in evaluating asthma treatment efficacy. However, few studies used the same setups and conditions with different drug forms or devices. Therefore, in this study, the amounts of budesonide deposited in each stage of the Marple-type Cascade Impactor were evaluated by delivering the drug as a DPI or an inhalant suspension (jet-type NE-C28 or mesh-type NE-U22 nebulizer). Particle size distributions and bronchial deposition rates were evaluated. This enabled the comparison of the relative efficacies of the various therapies and further identified the therapy that delivered the highest drug doses to the airway obstruction region.

## 2. Materials and Methods

### 2.1. Weibel's Anatomy Data

**Figure 1:**
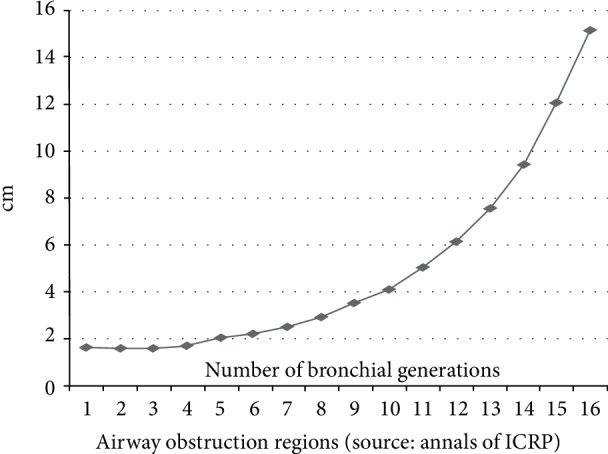
Diameter of bronchial generations. First to fourth bronchial generation diameters were calculated on the basis of the total cross-sectional area of a single duct and were markedly narrow in all bronchial generations.

Weibel anatomy data [3] (Table 1), which show the relationships between total cross-sections (Figure 1) and bronchial generations in bronchial asthma, were used to identify the airway obstruction regions. Weibel's respiratory tree model was first reported in 1963 and adopted in the ICRP respiratory tract textbook [4]. It is still cited in papers published in the twenty-first century.

Obstructed lung regions were determined from the Weibel anatomy data. In this model, the airway repeatedly bifurcates. The airways from the 1^st^ to 4^th^ generations are called bronchi.

Those from the 5^th^ to 16^th^ generations are known as bronchioles. The airways from the 17^th^ to 19^th^ generations are referred to as respiratory bronchioles, while those from the 20^th^ to 23^rd^generations are the alveolar ducts and sacs. The luminal walls of the 17^th^ and more distal generations consist of the alveolar wall. Therefore, the airways from the 1^st^ to 16^th^ generations are known as bronchioles. Those from the 8^th^ to 16^th^ generations which are <2 mm in diameter are known as small airways or peripheral bronchi. These initially bifurcate by reducing their internal diameter at a rate of 1/√2 but, the tapering rate gradually decreases in more distal areas. Therefore, total cross-sectional area increases with airway distality. Compared with the tracheal cross-sectional area (2.54 cm^2^), those of the 4^th^, 8^th^, 11^th^, and 16^th^ generations are 0.98x, 2.7x, 11.3x, and 70.8x in size, respectively.

### 2.2. Total Cross Section and Obstructive Region

Airway diameters were calculated using one duct from the total Weibel's cross-section (Figure 1). Figure 1 shows that the diameters of the 1^st^–4^th^ bronchial generations are mainly small and constitute a physiological airway obstruction region. The airways of the 1^st^–4^th^generations' obstruction sites serially bifurcate and are the narrowest according to the calculations of one duct from the total cross-sectional area. It was, therefore, considered that the asthmatic airway in Figure 1 showed the diameter of one duct based on the total cross-section obstruction region when it was localized to the 1^st^–4^th^ bronchial generations. It is obvious from Figure 1, that the airway of 1^st^–4^th^ generations is the most narrow of the total airways in the lung. Eosinophilic inflammation [5] occurred in all lung airways, but was limited in the airway obstruction region.

### 2.3. Aerosol Particle Size Deposits on Obstructive Region and European Standardization Committee

**Figure 2:**
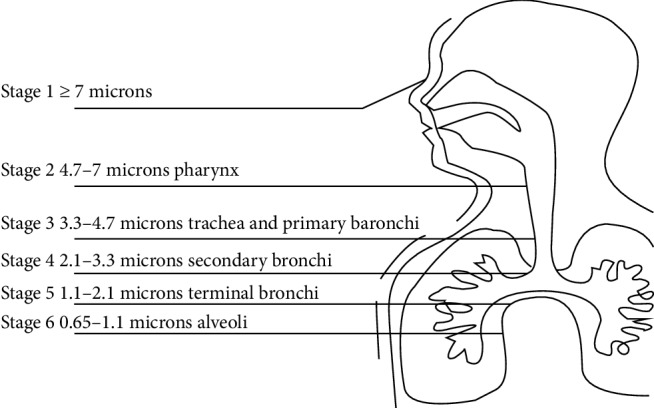
Andersen Instruments Inc. samplers simulating the human respiratory system. Particles 3.3–4.7 *µ*m in diameter were deposited in the trachea and primary bronchi (1^st^ to 4^th^ bronchial generation). On Andersen Cascade Impactor with 6 stages.

The sizes of the particles deposited in the airway obstruction regions (1^st^–4^th^ bronchial generations) are determined from the European Standardization Committee (CES) (Table 2) and the Cascade Impactor (Figure 2) data. The aerosol particle sizes and the pulmonary deposition areas were obtained from the European Standardization Committee data (CES) (EN 13544-1) [6] (Table 2). The Aerosol particle sizes were defined in terms of mass median aerodynamic diameter (MMAD) and geometric standard deviation (GSD). The consensus was that the efficacious aerosol particle diameter for asthmatic inhalation treatment is in the range of 2–6 *µ*m (Table 2).

### 2.4. Andersen Instrument (Cascade Impactor Manual Data)

The regions of trachea and primary bronchi are almost matched with 1^st^–4^th^ bronchial generations. On trachea and primary bronchi, particles of 3.3–4.7 *µ*m are deposited. The MMAD on Andersen Instrument is 4.0 *µ*m as normal distribution, which quite matched with the MMAD of the European Standardization Committee. In the next step, we chose the particle distribution of CES, because the data of CES are supported by thousands of published papers.

### 2.5. Particle Size Analyzed by Cascade Impactor

**Figure 3:**
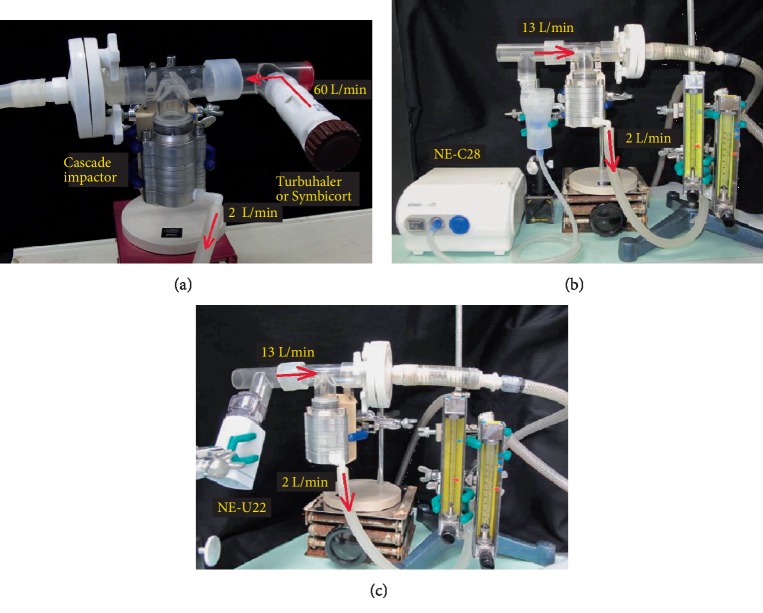
The sizes of the particles, deposited in the airway obstruction regions, generated by the (a) Turbuhaler or Symbicort (DPI), (b) NE-U22 (mesh nebulizer), and (c) NE-C28 (jet nebulizer). All particle sizes were measured using the Cascade Impactor. This connective method and air flow speed are basic style of Marple 8 stage cascade impactor.

The Cascade Impactor (Andersen Instruments, Inc.) is used mainly to analyze the atmosphere, [7] but it is also now used to simulate the lung airway (Figure 2). The Marple Personal Cascade Impactor 298 [8, 9] was used (Figures 3(a)–3(c)). Its specifications are as follows: cutoffs at impactor stages 1–7, and 8: 21.3, 14.8, 9.8, 6.0, 3.5, 1.55, 0.93, and 0.52 *µ*m, respectively, flow rate: 2 L/min (nominal), 0.5–5 L/min (lower and upper limits), and 1–3 L/min (recommended). The device is constructed from precision-machined aluminum and the impactor stages are nickel plated. Stages 7 and 8 have holes rather than slits to simulate real bronchial bifurcations. At the more distal sites of the airway model, the total cross-sectional area of the slits decreases and the flow velocity increases. For the bronchial bifurcations, however, the total cross-sectional area increases at the more distal sites, reaching a maximum at the terminal alveolar generation where the flow velocity falls to zero.

### 2.6. High-Performance Liquid Chromatography

For each Cascade Impactor stage, the budesonide trapped on the GF/A filters was assayed by HPLC (Shimadzu LC-10ADVP; Shimadzu Corp., Tokyo Japan) under the following conditions.

1. Column: Shim-pack VP-ODS 150 mm × 4.6 mm.
2. Mobile phase: Methanol : acetic acid 0.1% (7 : 3).
3. Retention time: 8 min.

The airway deposition rate was evaluated according to a particle size spectrum using the Cascade Impactor.

### 2.7. MMAD and Particle Size Distribution of Dry Powder and Suspension of Budesonide

**Figure 4:**
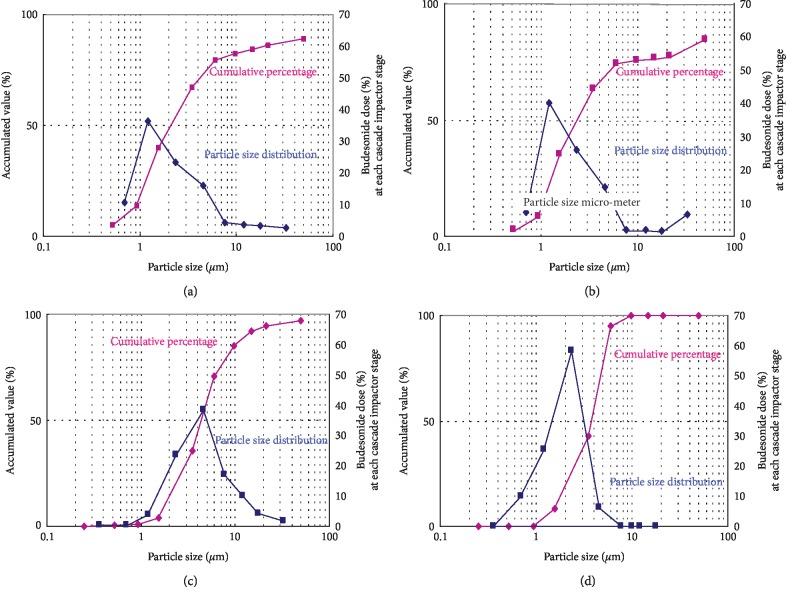
(a) Mass median aerodynamic diameters (MMAD) of the particle size distribution. Turbuhaler, 200 *µ*g; MMAD, 2.20 *µ*m, (b) Mass median aerodynamic diameters (MMAD) of the particle size distribution. Symbicort Turbuhaler, 60 DPI; MMAD, 2.60 *µ*m, (c) Mass median aerodynamic diameters (MMAD) of the particle size distribution. NE-U22; budesonide suspension, 250 *µ*g/mL; MMAD, 4.52 *µ*m, and (d) Mass median aerodynamic diameters (MMAD) of the particle size distribution. NE-C28; budesonide suspension, 250 *µ*g/mL; MMAD, 3.84 *µ*m.

The tracheobronchial regions correspond to the size of 1^st^–4^th^ bronchial generations. Therefore, from CES, particles 2–6 *µ*m in diameter are expected to deposit in the airway obstruction regions. The mass median aerodynamic diameters (MMAD) of the particle size distribution were 2.20 *µ*m for the Turbuhaler (DPI) (Figure 4(a)), 2.60 *µ*m for the Symbicort (DPI) (Figure 4(b)), 4.52 *µ*m for the mesh-type NE-U22 suspension inhalant (Figure 4(c)), and 3.84 *µ*m for the jet-type NE-C28 suspension inhalant (Figure 4(d)) (Table 1).

The experimental conditions were applied based on the European standard (EN13544-1 2007) Annex CC. According to EN 1354-1(2007), the ambient condition shall be 23 ± 2, Relative humidity 45–75% Rh. Thus the data were obtained at 22, 50% Rh.

## 3. Discussion

### 3.1. Deposition Rate on Obstructive Region by Dry Powder and Suspension of Budesonide

All these measurements were within the 2–6 *µ*m range. Therefore, the particles should have been deposited in the bronchi. All four distribution patterns were unimodal. The percentages of particles within the 2–6 *µ*m diameter range were 33.0% for the Turbuhaler, 32.0% for the Symbicort, 59.0% for the mesh-type NE-U22 suspension, and 78.0% for the jet-type NE-C28 suspension (Table 1). The percentages of particles generated by the electric nebulizers within the 2–6 *µ*m diameter range were higher than those generated by the DPIs. Differences in budesonide deposition rate and distribution were compared for suspension- (jet and mesh nebulizers) and dry powder (Pulmicort and Symbicort) inhalation methods using the Cascade Impactor. The data derived from the Cascade Impactor describe the relationship between aerosol particle size and bronchial generation. The 4.7–7.0, 3.3–4.7, 2.1–3.3, 1.1–2.1, and 0.6–1.1 deposition sites correspond to the pharynx, trachea, and primary bronchi, secondary bronchi, terminal bronchi, and alveoli, respectively. The bronchial obstruction site consisting of the 1^st^ to 4^th^ generations according to Weibel's anatomy data nearly aligns with the trachea and the primary obstruction site (deposition diameter range: 3.3–4.7 *µ*m). In these studies, the data from the two sources were compared. The 2–6 *µ*m diameter range was taken as the optimal deposited particle size in the airway obstruction region in bronchial asthma because it is corroborated by many literature citations.

### 3.2. Particle Size in Current pMDI

The pMDIs react with CFCs and form CFC-pMDIs which are then converted to HFA-pMDIs [10]. HFA + alcohol-pMDI is a super-small aerosol which can effectively reach and treat small airway obstructions. The MMADs of beclomethasone pMDI (Qvar, Sumitomo Dainippon Pharma Co., Ltd., Tokyo, Japan) and ciclesonide pMDI (Alvesco, Teijin Pharma, Tokyo, Japan) are 1.1 *µ*m and 0.9 *µ*m by each statement of virtues of a medicine, respectively.

Certain pulmonologists recommend that super-small steroid particles are efficacious in the management of small airway (bronchial diameter <2 mm) obstruction [11, 12]. However, the total cross sectional areas of the 8^th^ (Weibel anatomy data; (bronchial diameter 1.86 mm; small airway; calculated from the total cross section as a single duct)), 9^th^(1.54 mm), 10^th^ (1.3 mm), and 11^th^ (1.09 mm) bronchial generations were 6.95 cm^2^, 9.65 cm^2^, 13.4 cm^2^, and 19.6 cm^2^, respectively, which are far larger than the airway obstruction regions (<2.5 cm^2^). Therefore, small airway obstruction may, in fact, be absent in bronchial asthma, and the particle diameters of beclomethasone- and ciclesonide pMDI are too small to enable these drug forms to be efficacious for the treatment of this condition. In this study, the most major defect point affected is only inspiratory, not expiratory phase by the Cascade Impactor. About expiratory phase, no examinations could be performed. Bronchial asthma is an expiratory distress disease, mechanism of expiratory distress must be examined. According to those examinations performed, the identity of super small aerosol released pMDI may be useful to small airway obstruction.

### 3.3. Estimation of Inhaled Steroid Efficacy by One Factor as Particle Size is Difficult

In theory, the cutoff diameters of the particles deposited by inertial collision are determined at each stage. Particles 2–6 *µ*m in diameter are deposited in the bronchi/bronchioles. Particles are deposited on a Cascade Impactor primarily by inertia, whereas deposition which actually occurs by diffusion or sedimentation in the terminal airway of a living lung is not considered. Consequently, an airway tree model which more nearly approximates living lungs is required.

The percentage of particles <2 *µ*m in diameter deposited in the alveoli was 46.0% for the Turbuhaler, 42.0% for the Symbicort, 11.0% for the mesh-type NE-U22 suspension, and 17% for the jet-type NE-C28 suspension. The percentages of particles <2 *µ*m in diameter generated by the DPI (Pulmicort and Symbicort) were higher than those generated by the mesh- and jet-type electric nebulizers.

Evaluation of the efficacy of steroid inhalation for asthma treatment is very difficult. Relative to the cromoglycate inhalation therapy, the Pulmicort suspension has greater clinical efficacy [13].

However, certain reports [14–16] describe differences in clinical efficacy between Symbicort and ADVAIR. Nevertheless, these assessments are challenging because clinical symptom scores are unstable and the clinical visit continuity is very low [17]. To this end, we devised a new asthma inhalation therapy effectiveness evaluation system based on aerosol particle size and distribution. At any rate, the final evaluation must nonetheless be based on the degree of clinical improvement.

### 3.4. On Budesonide Inhalation Therapy, Nebulizer Inhalation Treatment by Pulmicort Suspension is the Best Method (Table 3)

The Cascade Impactor was previously used to assess cromoglycate efficacy (DPI, pMDI, and suspension) [18]. In the present study, the effectiveness of budesonide (DPI and suspension) was evaluated. The mass median aerodynamic diameter (MMAD) of particle size distribution was 2.20 *µ*m for the Turbuhaler (DPI), 2.60 *µ*m for the Symbicort (DPI), 4.52 *µ*m for the mesh-type NE-U22 inhalant, and 3.84 *µ*m for the jet-type NE-C28 inhalant (Figure 3). Particles within the 2–6 *µ*m diameterrange were deposited in the bronchi. The distribution pattern was unimodal for the other dosage forms. The percentages of particles 2–6 *µ*m in diameter generated by the Turbuhaler, Symbicort, mesh-type NE-U22 suspension, and jet-type NE-C28 suspension were 33.0%, 32.0%, 59.0%, and 78.0%, respectively (Table 2). The electric jet nebulizer delivered ~2.36x more drug to the bronchi/bronchioles than the Turbuhaler. The normal budesonide dose delivered with the Pulmicort 200 Turbuhaler is 400 *µ*g/d; whereas, with the Pulmicort Suspension it is 1,000 *µ*g/d. Based on deposition rates of 33% with the Turbuhaler and 78% with the NE-C28 jet nebulizer suspension, the rates of budesonide dose deposition to the airway obstruction area are 132 *µ*g/d and 780 *µ*g/d, respectively. In fact, the rate of budesonide deposition by NE-C28 into the bronchial obstruction area is 5.9x higher than that delivered by the Turbuhaler. The results of the present study suggest that NE-C28 inhalation is the most efficacious method of delivering budesonide for asthma therapy.

Differences in the diameters of the inhaled budesonide particles result in variable degrees of improvement in the clinical symptoms of bronchial asthma. We investigated reports on comparative variations in bronchial asthma mitigation in response to the inhalation of the dry powder and suspension forms of budesonide. Nevertheless, no studies were found comparing clinical improvements using different budesonide dosage forms and application devices. When equivalent budesonide doses in DPI and suspension form (NE-C28) are administered, the latter has an obvious advantage in terms of clinical bronchial asthma improvement because it deposits 2.36x more budesonide than the former. Budesonide inhalation routes, application devices, and doses merit further research.

Currently inhaled asthma drugs have a particle diameter range of 0.9–10 *µ*m. Compared the volume or gravity of big and small aerosols in current inhaled drugs, the big aerosol of volume or gravity is reached to 1000 times as cubic of diameter scale. A future study objective is to optimize the amount of inhalation drug which reaches the airway obstruction region including small airway (16, 17) in bronchial asthma.

## 4. Conclusion

Among the various dosage forms of budesonide, the NE-C28 suspension may deposit ≤2.36x more drug in the bronchial obstruction region than the Pulmicort Turbuhaler (DPI). The NE-C28 suspension inhalation therapy, then, may be the most efficacious type of budesonide inhalation therapy for bronchial asthma. The particle size (MMAD: 3.84 *µ*m) and distribution (2–6 *µ*m: 78%) obtained for the jet-type nebulizer suspension may have the greatest access to the airway obstruction region in bronchial asthma. The particle size (MMAD: 2.20) and distribution (<2 *µ*m: 46%) obtained using the Turbuhaler (DPI) may be best suited for the treatment of peripheral lung diseases such as eosinophilic pneumonia, and COPD (chronic 260 obstructive pulmonary disease).

## Figures and Tables

**Table 1:** Dimensions of human model A (Source: annals of the ICRP). Weibel anatomy data. According to the bronchial generation, which shows number per generation, diameter, length, total cross section and total volume.

	Generation	Number per generation	Diameter (cm)	Length (cm)	Total cross-section (cm^2^)	Total volume (cm^3^)
Broncus	0	1	1.8	12	2.54	30.5

Primary bronchi	1	2	1.22	4.76	2.33	11.25
	2	4	0.83	1.9	2.13	3.97
	3	8	0.56	0.76	2	1.52
	4	16	0.45	1.27	2.48	3.46

Bronchiole	5	32	0.35	1.07	3.11	3.3
	6	64	0.28	0.9	3.96	3.53
	7	128	0.23	0.76	4.1	3.85
	8	256	0.186	0.64	6.95	4.45
	9	512	0.154	0.54	9.65	5.17
	10	1024	0.13	0.46	13.4	6.31
	11	2048	0.109	0.39	19.6	7.56
	12	4096	0.095	0.33	28.8	9.82
	13	8192	0.082	0.27	44.5	12.45
	15	16384	0.074	0.23	69.4	16.4
	15	32768	0.066	0.2	113	21.7
	16	65536	0.06	0.165	180	29.7

Respiratory bronchiole	17	131072	0.054	0.141	300	41.8
	18	262144	0.05	0.117	534	61.1
	19	524288	0.047	0.099	944	93.2

Alveolus	20	1048576	0.045	0.083	1600	139.5
	21	2097152	0.043	0.076	3220	224.3
	22	4194304	0.041	0.059	5880	350
	23	8388608	0.041	0.05	11800	591

**Table 2:** European Standardization Committee gives a definition that particle with 2-6 *µ*m diameter deposits on tracheobronchi.

Deposition region	Particle diameter (*µ*m)
Upper airways	>5
Tracheobronchi	2–6
Alveoli	0.5–3

European 135 standardization committee IEN 44-1 : 2007 (E). Aerosol particles of diameter: >5 *µ*m will deposit in the upper airways; 2–6 *µ*m will deposit in the tracheobronchi; 0.5–3 *µ*m will deposit in the alveoli. These data were corroborated by >1,000 published papers and books.

**Table 3:** Budesonide particle size by application device per Cascade Impactor. On budesonide inhalation therapies NE-C28 and NE-U22 have advantage of 78% and 59% with 2 < particle size < 6 *µ*m respectively. These data are double deposit percentage of Turbohaler and Symbicort dry powder.

*N* = 3	Mean	Turbo haler	Symbicort	NE-U22	NE-C28
MMAD(*µ*m)	Mean	2.20	2.60	4.52	3.84
	SD	0.47	0.68	0.37	0.15
<6 *µ*m %		80	74	71	95
2 < particle size < 6 Μm%		33	32	59	78
<2 *µ*m%		46	42	11	17

## Data Availability

The data used to support the findings of this study are available from the corresponding author upon request.

## Abbreviations

CFC: Chlorofluorocarbon
COPD: Chronic obstructive pulmonary disease
DPI: Dry powder inhalation
HFA: Hydrofluoroalkane
MMAD: Mass median aerodynamic diameter
pMDI: Pressurized metered dose inhaler.

## Supplementary Materials

Supplementary Material In the present study, we compared different budesonide inhalation therapy dosage forms and application devices. First to fourth generation tracheal bronchi; particle size deposited: 2–6 *µ*m; deposition rate: 78% (NE-C28 suspension).

## References

[1] Murayama N., Doi S., Inoue T., Takamatsu I., Kameda M., Takeda K. (2018). Inhaled steroid inhibits development of total and mite IgE. *Immunological Medicine*.

[2] Murayama N., Kameda M., Takamatsu I., Inoue T., Doi S., Toyoshima K. (1996). Difference of lung deposition rate of disodium cromoglycate (DSCG) among three kinds of electric nebulizer. *Arerugi*.

[3] Weibel E. R. (1963). Principles and methods for the morphometric study of the lung and other organs. *Laboratory Investigation; A Journal of Technical Methods and Pathology*.

[4] Gehr P., Annexe A. (1994). Anatomy and morphology of the respiratory tract. *Annals of the ICRP*.

[5] Hamid Q. (2012). Pathogenesis of small airways in asthma. *Respiration*.

[6] (2007). Respiratory therapy equipment part 1. Nebulizing systems and their components.

[7] Wu Y. H., Vincent J. H. (2007). A modified Marple-type Cascade Impactor for assessing aerosolparticle size distributions in workplaces. *Journal of Occupational and Environmental Hygiene*.

[8] Chen M. R., Tsai P. J., Chang C. C., Shih T. S., Lee W. J., Liao P. C. (2007). Particle size distributions of oilmists in workplace atmospheres and their exposure concentrations to workers in a fastener manufacturing industry. *Journal of Hazardous Materials*.

[9] Series 290 Instruction Manual (2009). Marple Personal Cascade Impactors. https://assets.thermofisher.com/TFS-Assets/LSG/manuals/EPM-manual-290.pdf.

[10] Leach C. L., Davidson P. J., Boudreau R. J. (1998). Improved airway targeting with the CFC-free HFA-beclomethasone metered-dose inhaler compared with CFC-beclomethasone. *European Respiratory Journal*.

[11] Husemann K., Haidl P., Kroegel C., Voshaar T., Kohlhaufl M. (2012). Lung function diagnostics for the small airways. *Pneumologie*.

[12] van den Berge M., ten Hacken N. H. T., van der Wiel E., Postma D. S. (2013). Treatment of the bronchial tree from beginning to end: targeting small airway inflammation in asthma. *Allergy*.

[13] Leflein J. G., Szefler S. J., Murphy K. R. (2002). Nebulized budesonide inhalation suspension compared with cromolyn sodium nebulizer suspension for asthma in young children: results of a randomized outcomes trial. *Pediatrics*.

[14] Stanford R. H., Averell C., Parker E. D., Blauer-Peterson C., Reinsch T. K., Buikema A. R. (2019). Assessment of adherence and asthma medication ratio (AMR) for a once-daily and twice-daily inhaled corticosteroid/long acting beta agonist (ICS/LABA) for asthma. *The Journal of Allergy and Clinical Immunology: In Practice*.

[15] Tashkin D. P., Moore G. E., Trudo F., DePietro M., Chipps B. E. (2016). Assessment of consistencyof fixed airflow obstruction status during budesonide/formoterol treatment and its effects on treatment outcomes in patients with asthma. *The Journal of Allergy and Clinical Immunology: In Practice*.

[16] Tunceli O., Williams S. A., Kern D. M. (2014). Comparative effectiveness of budesonide-formoterol combination and fluticasone-salmeterol combination for asthma management: a United States retrospective database analysis. *The Journal of Allergy and Clinical Immunology: In Practice*.

[17] Hayashida M., Murayama N., Toyoshima K. (2012). Persistence rate for clinic visit in children with asthma after initiating controller therapy. *Arerugī*.

[18] Murayama N., Asai K., Murayama K., Kitatsuji C., Doi S. (2017). Deposition dosages of threecromolyn forms by Cascade Impactor. *Journal of Drug Delivery*.

