# Supplementary material for: Dry Powder and Budesonide Inhalation Suspension Deposition Rates in Asthmatic Airway-Obstruction Regions
Source: J Drug Deliv. 2019 Nov 18;2019:3921426. doi: 10.1155/2019/3921426 (PMC6885778; doi:10.1155/2019/3921426)
Supplement: Supplementary Material — In the present study, we compared different budesonide inhalation therapy dosage forms and application devices. First to fourth generation tracheal bronchi; particle size deposited: 2–6 µm; deposition rate: 78% (NE-C28 suspension). [file 3921426.f1.pdf]

Graphical Abstract

Weibel's Anatomy Data

Total cross section from 1st to 4th bronchial generations: no change; narrowest regions of all bronchial generations.

Bronchial generations 1-4 are in the airway obstruction region

European Standardization Committee

Relationship between aerosol size and deposit regions by CEM. Corroborates or is corroborated by > 1,000 papers and books.

Bronchial generations 1-5 correspond to the Tracheobronchi where particles 2-6 μm in diameter can be deposited.

Cascade Impactor

For the Pulmicort DPI, 33% of all particles were 2-6 μm in diameter.

For the Pulmicort suspension by NE-C28, 78% of all particles were 2-6 μm in diameter.

% of particles 2-6 μm in diameter for NE-C28 was 2.36 × that for DPI.
